# Combined Delivery of Paclitaxel and Tanespimycin via Micellar Nanocarriers: Pharmacokinetics, Efficacy and Metabolomic Analysis

**DOI:** 10.1371/journal.pone.0058619

**Published:** 2013-03-07

**Authors:** Usha Katragadda, Wei Fan, Yingzhe Wang, Quincy Teng, Chalet Tan

**Affiliations:** 1 Cancer Nanomedicine Laboratory, Department of Pharmaceutical Sciences, College of Pharmacy and Health Sciences, Mercer University, Atlanta, Georgia, United States of America; 2 Department of Pharmaceutical and Biomedical Sciences, College of Pharmacy, University of Georgia, Athens, Georgia, United States of America; Argonne National Laboratory, United States of America

## Abstract

**Background:**

Despite the promising anticancer efficacy observed in preclinical studies, paclitaxel and tanespimycin (17-AAG) combination therapy has yielded meager responses in a phase I clinical trial. One serious problem associated with paclitaxel/17-AAG combination therapy is the employment of large quantities of toxic organic surfactants and solvents for drug solubilization. The goal of this study was to evaluate a micellar formulation for the concurrent delivery of paclitaxel and 17-AAG *in vivo*.

**Methodology/Principal Findings:**

Paclitaxel/17-AAG-loaded micelles were assessed in mice bearing human ovarian tumor xenografts. Compared with the free drugs at equivalent doses, intravenous administration of paclitaxel/17-AAG-loaded micelles led to 3.5- and 1.7-fold increase in the tumor concentrations of paclitaxel and 17-AAG, respectively, without significant altering drug levels in normal organs. The enhanced tumor accumulation of the micellar drugs was further confirmed by the whole-body near infrared imaging using indocyanine green-labeled micelles. Subsequently, the anticancer efficacy of paclitaxel/17-AAG-loaded micelles was examined in comparison with the free drugs (weekly 20 mg/kg paclitaxel, twice-weekly 37.5 mg/kg 17-AAG). We found that paclitaxel/17-AAG-loaded micelles caused near-complete arrest of tumor growth, whereas the free drug-treated tumors experienced rapid growth shortly after the 3-week treatment period ended. Furthermore, comparative metabolomic profiling by proton nuclear magnetic resonance revealed significant decrease in glucose, lactate and alanine with simultaneous increase in glutamine, glutamate, aspartate, choline, creatine and acetate levels in the tumors of mice treated with paclitaxel/17-AAG-loaded micelles.

**Conclusions/Significance:**

We have demonstrated in the current wok a safe and efficacious nano-sized formulation for the combined delivery of paclitaxel and 17-AAG, and uncovered unique metabolomic signatures in the tumor that correlate with the favorable therapeutic response to paclitaxel/17-AAG combination therapy.

## Introduction

Combination therapy with two or more anticancer drugs is the cornerstone of cancer therapy. However, it remains a major challenge to achieve safe and efficient delivery of drugs to the tumor. Nano-sized drug carriers are emerging drug delivery systems for cancer therapy because of their unique ability to prolong the drug circulation times in blood, and promote drug accumulation in the tumor via the enhanced permeability and retention (EPR) effect [1]. With sizes usually ranging between 10–200 nm, nanoparticles are restricted from normal vasculature (1–2 nm fenestrations in most healthy tissues); they preferentially extravasate and accumulate at the tumor site owing to the leaky tumor vasculature (usually >100 nm fenestrations) and the impaired lymphatic drainage in solid tumors [2]. Although a large number of nanoparticulate delivery systems being developed over the past two decades, most research efforts focus on single drug encapsulation, whereas delivering multiple drugs with a single delivery carrier remains to be explored [3]−[6].

Heat shock protein 90 (Hsp90) is a highly conserved chaperon protein required for conformational maturation, activation and stability of a myriad of client proteins, many of which are oncogenic kinases and transcription factors that are vital in mediating oncogenic addition and tumor cell survival [7]. Hsp90 is recognized as an important drug target for cancer therapy with a number of Hsp90 inhibitors currently in clinical trials. Tanespimycin (17-AAG) was the first Hsp90 inhibitor to be evaluated clinically as a monotherapy and in combination with other anticancer drugs. However, the development of 17-AAG was hampered by its poor water solubility. In a phase I clinical trial, combination therapy of 17-AAG with paclitaxel yielded meager responses in advanced cancer patients [8]. The limited efficacy is at least in part attributed to inadequate inhibition of the targeted proteins, owing to the suboptimal dosing level and frequency of the drugs. The dose-limiting toxicities of paclitaxel/17-AAG combination therapy are not only caused by the drugs alone, they are also attributable to the large volume of toxic organic surfactants and solvents (Cremophor EL/ethanol for paclitaxel; DMSO/egg phospholipid for 17-AAG) used to solubilize these two poorly water-soluble drugs. Cremophor EL induces hypersensitivity reactions in patients, which necessitates the pretreatment with corticosteroids and antihistamines, whereas DMSO causes serious adverse effects including hepatotoxicity, cardiotoxicity, nausea, vomiting and malodor.

**Figure 1:**
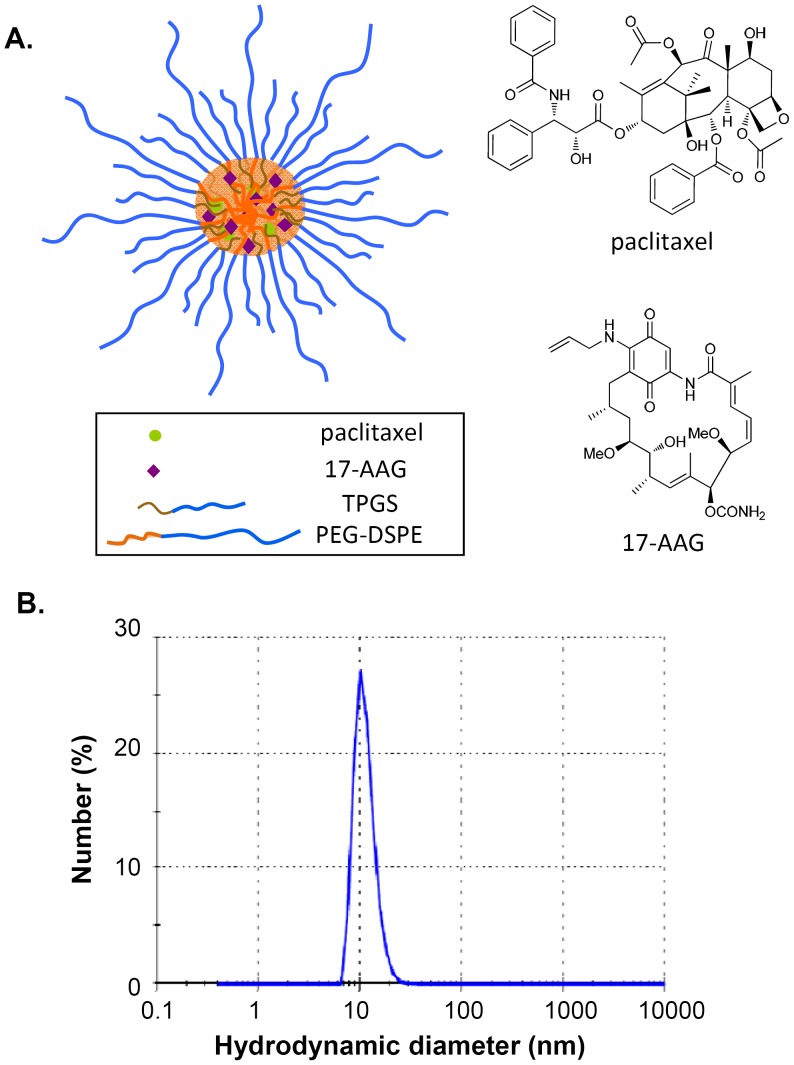
Paclitaxel/17-AAG-loaded PEG-DSPE/TPGS mixed micelles. A, the structural scheme of the dual drug-loaded micelles. B, the hydrodynamic diameter of the dual drug-loaded micelles. Number (%) shows among the total number of the counted particles in a sample, the percentage of the number of the particles within each size class. The result shows representative data obtained from 3 independent measurements.

One promising approach to overcome the severe toxicities caused by the toxic organic excipients and achieve effective paclitaxel/17-AAG combination therapy is to formulate these drugs into nano-sized drug carriers. Composed of biocompatible and biodegradable amphiphilic copolymers, we have previously shown that PEG-DSPE/TPGS mixed micelles can concomitantly encapsulate therapeutic levels of paclitaxel and 17-AAG into the lipophilic micellar core without the inclusion of any organic solvent [9]. In the current study, we demonstrated that in comparison with the drugs in their free forms, paclitaxel/17-AAG-loaded micelles (Fig. 1A) markedly prolonged the circulation times of both drugs and enhanced the drug accumulation in the tumor, leading to substantially improved anticancer efficacy in a human ovarian tumor mouse model. By incorporating indocyanine green (ICG) into PEG-DSPE/TPGS mixed micelles, an FDA-approved near infrared (NIR) imaging agent, we were able to track the real-time distribution of the ICG-labeled micelles in the body and particularly in the tumor, which illustrates the theranostic potential of this delivery system. Importantly, by analyzing the tumor metabolome using proton nuclear magnetic resonance (1H NMR) spectroscopy, we uncovered for the first time unique metabolomic signatures in the tumor that correlate with the favorable therapeutic response to paclitaxel/17-AAG combination therapy.

## Materials and Methods

### Ethics Statement

All animal procedures were performed in strict accordance with the Guide for the Care and Use of Laboratory Animals of the National Institutes of Health. The protocol was approved by the Institutional Animal Care and Use Committee at Mercer University (Approval Number: A1105007).

### Chemicals, Reagents and Cell Line

Paclitaxel and 17-AAG were purchased from LC Laboratories (Woburn, MA). 1,2-Distearoyl-*sn*-glycero-3-phosphoethanolamine-*N*-[methoxy(polyethylene glycol)-2000] (PEG-DSPE) was purchased from Corden Pharma (Cambridge, MA). D-α-tocopheryl polyethylene glycol 1000 succinate (TPGS) was obtained from Eastman Chemical Company (Kingsport, TN). ICG was purchased from Tokyo Chemical Industry Company (Tokyo, Japan). Human ovarian cancer SKOV-3 cells were obtained from the American Type Culture Collection (ATCC, Manassas, VA) and grown in DMEM medium (Invitrogen, Carlsbad, CA) with 2 mM L-glutamine, which was supplemented with 10% (v/v) fetal bovine serum, 100 units/ml penicillin G and 100 µg/ml streptomycin. The cells were maintained at 37°C with 5% CO_2_ in a humidified incubator.

### Preparation of Paclitaxel/17-AAG-loaded Micelles

A solvent evaporation method was employed to prepare the drug-loaded micellar nanocarriers as reported previously [9]. In a typical preparation, 0.124 mg paclitaxel, 0.234 mg 17-AAG, 5.6 mg PEG-DSPE and 5.7 mg TPGS were dissolved in 1 ml chloroform in a 25-ml round-bottom flask, which was rotor-evaporated to dryness at room temperature to form a homogenous thin drug-polymer film. The resulting thin film was further dried overnight under vacuum to remove any residual solvent. The film was then hydrated with 100 µl 10 mM HEPES-buffered saline (HBS, pH 7.4) while vigorous vortexing for 5 min at room temperature. The resulting mixture was centrifuged at 12,000 g for 5 min to remove any undissolved drugs/polymers, yielding clear micellar dispersion. The micelles were formed efficiently with minimal precipitation. The resulting micellar solution was composed of 1.45 mM paclitaxel, 4.0 mM 17-AAG, 20 mM PEG-DSPE and 40 mM TPGS. The loading concentrations of paclitaxel and 17-AAG in the micelles, defined as the amount of the drugs in the resulting micellar solution per unit volume of HBS, were quantified by HPLC as described earlier [9]. Because the aqueous solubility of free paclitaxel and 17-AAG is merely 0.3 µM and 17 µM, respectively, which were less than 0.5% of the drug levels in the micellar solution, the obtained HPLC results herein reflected the concentrations of drugs loaded within PEG-DSPE/TPGS mixed micelles. The drug concentrations in the micellar solution were controlled by adding exact amount of drugs during the micelle preparation, and the loading efficiency of each drug was above 95%, which is defined as the ratio between the drug amount in the resulting micellar solution and the input drug amount. The hydrodynamic diameter of the micelles was evaluated by dynamic light scattering using a Nano Zetasizer (Malvern Instruments, UK) equipped with He-Ne laser (4 mW, 633 nm) light source and 90° angle scattered light collection configuration. Prior to the size measurement, the micelle sample was diluted in HBS (10 mM, pH 7.4) by 10 times and filtered through 0.2 µm membrane. The storage stability of the dual drug-loaded micelles was assessed by monitoring the drug concentrations and the micelle size following incubation at 37°C or 4°C for 4 weeks.

### Tumor Xenograft Mouse Model

Human ovarian cancer SKOV-3 cells (2 x 10^6^ in 0.1 ml matrigel/DMEM mixture) were implanted subcutaneously in each flank of 5–6 week-old female athymic nude mice (*nu/nu*, Charles River, Wilmington, MA). The tumor size was measured using a caliper, and the tumor volume was calculated as ½ × length × width^2^.

### Pharmacokinetic Study

**Figure 2:**
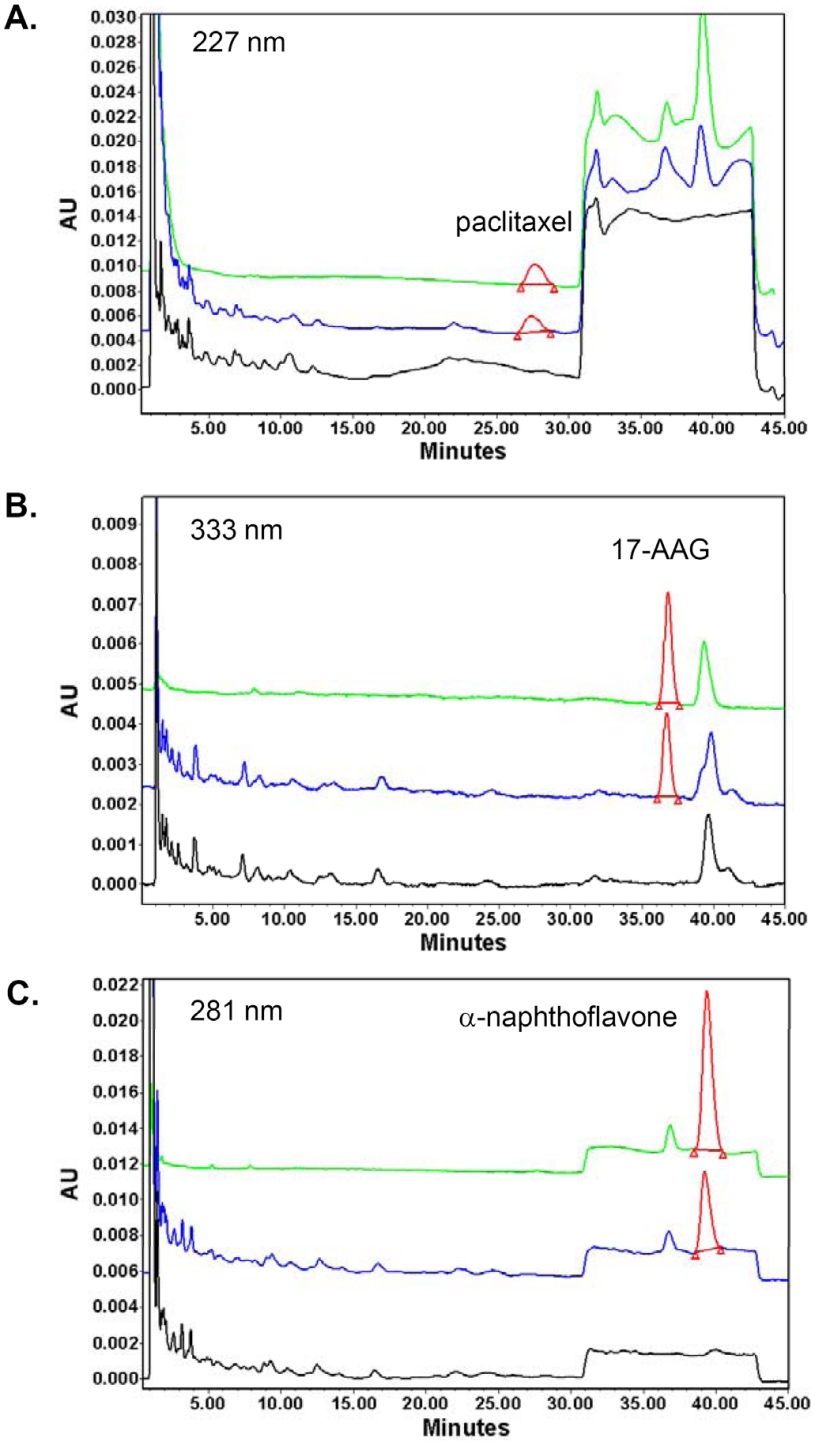
Simultaneous quantification of paclitaxel and 17-AAG by HPLC chromatography. Representative chromatograms of pure paclitaxel and 17-AAG (green trace), an extract of drug-spiked liver tissue (blue trace) and a blank liver extract (black trace) are shown at 221 nm (A), 333 nm (B) and 281 nm (C), while the peaks of interest at each detection wavelength are shown in red. The retention time of paclitaxel, 17-AAG and α-naphthoflavone was 27.6 min, 36.8 min and 39.3 min, respectively. The concentration of paclitaxel and 17-AAG was both 10 µM, and α-naphthoflavone was used at 20 µM as an internal standard.

Once SKOV-3 tumor volumes reached 100–300 mm^3^, the mice were randomized into study groups (4 mice per group). Sterilized via filtration through 0.2 µm membrane, the dual drug-loaded micelles were administered via the tail vein injection with paclitaxel and 17-AAG doses at 20 mg/kg and 37.5 mg/kg, respectively. For the free drug-treated group, the mice received the same combined doses of free paclitaxel and 17-AAG dissolved in DMSO. At 5, 10, 15, 30, 60, 120 and 240 min post injection, whole blood was collected via the retro-orbital bleeding. Normal organs (liver, lung, spleen, kidney and heart) and tumor tissues were harvested at 120 min. Following extraction using ethyl acetate, the concentrations of paclitaxel and 17-AAG in plasma and tissue homogenate samples were simultaneously determined by HPLC [10]. The HPLC system (Waters, Milford, MA) consisted of a Waters 2795 pump, a Phenomenex (Torrance, CA) C8 column (5 µm, i.d. 4.6 mm × 150 mm) and a Waters 996 photodiode array detector. α-Naphthoflavone was used as an internal standard. The detection wavelengths for paclitaxel, 17-AAG and α-naphthoflavone were 227 nm, 333 nm and 281 nm, respectively. To analyze plasma samples, an isocratic mobile phase of 25% (v/v) sodium phosphate buffer (25 mM, pH 3.0) with 10 mM triethylamine and 75% (v/v) methanol was used. For tissue homogenate samples, a gradient elution of A: sodium phosphate buffer and B: methanol (0–30 min 46% A, 30.01–40 min 28% A, 40.01–45 min 46% A) was used. The flow rate was 1.5 ml/min at 40°C. The assay methodology was established using blank plasma and tissue homogenate samples spiked with known drug concentrations. Following ethyl acetate extraction, paclitaxel, 17-AAG and α-naphthoflavone were completely resolved from endogenous interference peaks in all plasma and tissue extracts. Representative chromatograms of the pure drugs, an extract of drug-spiked liver tissue, and an extract of blank liver tissue at 227, 333 and 281 nm are shown in Fig. 2. The lower limits of quantification for paclitaxel and 17-AAG were 2.5 µM and 1 µM, respectively. The linear ranges for the standard curves of paclitaxel and 17-AAG were between 2.5–100 µM, and 1–20 µM, respectively. Non-compartmental analysis was performed to obtain pharmacokinetic parameters of paclitaxel and 17-AAG including the elimination half-life (t_1/2_), the total body clearance (CL_T_), and the apparent volume of distribution (V_D,ss_) using WinNonlin software (version 5.1, Pharsight, Sunnyvale, CA).

### *In vivo* NIR Fluorescence Imaging

ICG-labeled drug-loaded PEG-DSPE/TPGS mixed micelles were prepared using a dry film method as described above. The dry film consisting of paclitaxel, 17-AAG and copolymers was prepared as described above, which was subsequently hydrated with 100 µl sterile HBS (10 mM, pH 7.4) containing 0.16 mM ICG. The resulting ICG-labeled micelles was composed of 1.45 mM paclitaxel, 4.0 mM 17-AAG, 20 mM PEG-DSPE, 40 mM TPGS, and 0.16 mM ICG.

To confirm the incorporation of ICG into the micelles, the stability of ICG-labeled micelles was studied at 37°C. Freshly prepared free ICG solution in HBS at the identical concentration was used as a control. At 0, 2, 4, 8, 24 and 48 h, aliquots of the micellar or free ICG solution were analyzed for the fluorescence intensity at 800 nm using Odyssey imaging system (LI-COR Biosciences, Lincoln, NE). All samples were prepared and analyzed in triplicates.

ICG-labeled micelles were administered to SKOV-3 tumor-bearing mice via the tail vein injection at an ICG dose of 2 mg/kg. Freshly prepared free ICG solution in HBS was i.v. injected at the same dose to the control mice. Immediately following the injection (0 h) and at 1, 2, 4, 6, 24 and 48 h, the mice were anesthetized under a continuous flow of isoflurane/oxygen, and the spectral fluorescence signals of the whole body images (800 nm channel) were obtained using the Odyssey imaging system.

### Efficacy Study

SKOV-3 tumor-bearing mice with tumor volumes ranging between 50–150 mm^3^ were randomized into 3 groups with 6 mice each: (A) the untreated group; (B) the free drug-treated group; and (C) the micellar drug-treated group. For Groups B and C, the mice were i.v. dosed on days 0, 7 and 14 with the combined doses of paclitaxel (20 mg/kg) and 17-AAG (37.5 mg/kg) either as free drugs dissolved in DMSO, or the dual drug-loaded micelles. On days 3, 10 and 17, the mice in these two groups also received 17-AAG (37.5 mg/kg) as the free drug or the drug-loaded micelles. Tumor growth and the animal body weight were assessed twice-weekly. The mice were sacrificed on day 43, and tumors were excised and weighted. Each tumor was divided for immediate formalin fixation and flash freezing.

### Western Blot Analysis

Tumor tissue samples (approximately 20–25 mg per sample) were homogenized and lysed using RIPA buffer supplemented with protease and phosphatase inhibitors. Tumor lysates containing 35 µg of total protein were resolved onto 10% SDS-PAGE gels, and transferred onto nitrocellulose membranes. Blots were probed with antibodies for phosphorylated Akt (p-Akt), total Akt, p-GSK 3α/β and total GSK 3α/β, all of which were from Cell Signaling (Danvers, MA). Anti-β-actin antibody (Santa Cruz Biotechnology, Santa Cruz, CA) was used as a loading control. Subsequently, the blots were incubated with fluorescently labeled secondary antibodies (LI-COR) and visualized using the Odyssey imaging system. The protein levels were quantified using the densitometry function on the instrument, normalized to β-actin level of each sample.

### Histology and Immunohistochemistry

Formalin-fixed and paraffin-embedded tumor specimens of 3- to 4-µm thickness were sectioned and stained with hematoxylin and eosin (H&E) and anti-Ki-67 antibody (DAKO, Carpinteria, CA) following standard protocols. All histological and immunohistochemical staining was performed in the Pathology Core Laboratory of the Winship Cancer Institute at Emory University (Atlanta, GA).

### ^1^H NMR Analysis of Tumor Samples

Tumor tissue samples (approximately 5–10 mg per sample) were extracted using a dual phase extraction procedure as described previously [11]. For the current study, only the aqueous samples (the polar extracts) were used. Prior to NMR analysis, the extraction solvents were completely removed under vacuum. Each aqueous sample was then reconstituted in 230 µL of 100 mM sodium phosphate buffered deuterium oxide (pH 7.4) containing 20 µM sodium 3-(trimethylsilyl) propionate-2,2,3,3-d_4_ (as an internal reference, Sigma-Aldrich, St. Louis, MO).

All NMR spectra of the tumor extracts were collected at 20°C on a 600 MHz Agilent Inova spectrometer using a triple-resonance 3-mm probe (Santa Clara, CA). For one-dimensional NOESY spectra, 1024 transients were collected, using 2 seconds for acquisition time and a 2-second presaturation pulse for suppressing residual water resonance. Spectral assignments were made using the results of these experiments in conjunction with the previously reported chemical shift values and confirmed using Chenomx Profiler (Edmonton, Canada) [12].

### NMR Data Processing and Multivariate Data Analysis

Spectra were processed with 0.3-Hz line broadening followed by zero-filling to 64 k points using Mnova (MestreC Research, Santiago de Compostela, Spain). The spectra (0.50–10.00 ppm) excluding residual water resonance (4.7–5.1 ppm) were segmented into 0.005 ppm-wide bins and normalized by the total spectral area. The bins were mean-centered and Pareto-scaled prior to the statistical analyses using SIMCA-P+ (Umetrics Inc., Umea, Sweden). Principal components analysis (PCA) was used to screen for outliers in the data set and the orthogonal partial least-squares discriminant analysis (OPLS-DA) model was generated to visualize the impact of the drug treatments. The OPLS-DA model was validated within SIMCA-P+ using a training set (80% of the data) and a prediction set (the remaining 20% of the data). Student’s t-tests filtered difference NMR spectra were generated according to the procedure reported previously [13].

## Results

### Generation of Paclitaxel/17-AAG-loaded Micelles

We have previously reported that by precisely controlling the input amount of each drug, paclitaxel and 17-AAG can be concomitantly loaded into PEG-DSPE/TPGS mixed micelles at a fixed ratio [9]. At the loading concentrations of 1.45 mM paclitaxel and 4.0 mM 17-AAG, the dual drug-loaded micelles were formed efficiently with minimal precipitation. The average hydrodynamic diameter of paclitaxel/17-AAG-loaded PEG-DSPE/TPGS mixed micelles was about 11 nm with a unimodal and narrow distribution (Fig. 1B). These micelles were stable at both 4°C and 37°C for at least 4 weeks, evidenced by less than 5% decrease in the loading concentration of each drug, as well as the minimal change in the average diameter of the micelles.

### Pharmacokinetics of Paclitaxel/17-AAG-loaded Micelles in Tumor-bearing Mice

**Figure 3:**
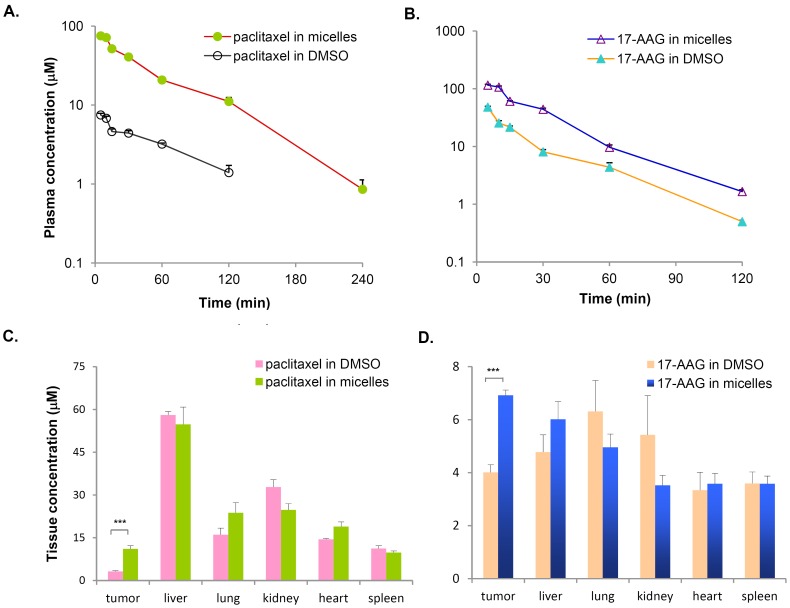
Pharmacokinetics of paclitaxel/17-AAG-loaded micelles in nude mice bearing human ovarian tumor SKOV-3 xenografts. The dual drug-loaded micelles were i.v. administered at the doses of 20 mg/kg paclitaxel and 37.5 mg/kg 17-AAG. For the free drug-treated group, the mice received the same combined doses of free paclitaxel and 17-AAG dissolved in DMSO. Each data point was the average+SE, n = 4 mice per group. A, the micellar formulation resulted in over 10-fold increase in paclitaxel concentrations in plasma. The plasma concentration of paclitaxel following the free drug administration was below the detection limit at 4 h. B, the micellar formulation resulted in over 3-fold increase in 17-AAG concentrations in plasma. The plasma concentration of 17-AAG was below the detection limit at 4 h for both groups. C, the micellar formulation caused a 3.5-fold increase of paclitaxel (***, p = 0.0001) in the tumor without significant affecting the drug distribution to normal organs. D, the micellar formulation caused a 1.7-fold increase of 17-AAG (***, p = 0.0005) in the tumor without significant affecting the drug distribution to normal organs.

To explore the therapeutic potential of paclitaxel/17-AAG-loaded micelles, we first undertook the task of characterizing the pharmacokinetics of micellar paclitaxel and 17-AAG. As a nano-sized drug delivery system, the drug-loaded micelles should ideally retain drug molecules for an extended period of time while circulating, extravasate into the tumor tissue via the EPR effect, and elevate the drug exposure in the tumor. To this end, we first analyzed plasma concentrations of paclitaxel and 17-AAG over a 4-h period following i.v. injection of the dual drug-loaded micelles. For the free drug-treated group, although Cremophor EL/ethanol is the clinically relevant solvent for paclitaxel, DMSO was chosen as the single delivery vehicle for both paclitaxel and 17-AAG for the following three reasons: (1) it was necessary to administer both drugs in a single tail vein injection, but we found the amount of Cremophor EL needed for simultaneously solubilizing paclitaxel and 17-AAG was lethal in mice; (2) DMSO was the adopted solvent for 17-AAG in the clinic, and could solubilize both drugs safely; (3) we aimed to evaluate the impact of PEG-DSPE/TPGS mixed micelles on the disposition of the loaded drugs in comparison with their free forms, whereas Cremophor EL is known to sequester paclitaxel in plasma and cause nonlinear plasma pharmacokinetics without increasing the tissue distribution of the drug. Compared with the combined free drugs at the equal doses, we observed that the micellar formulation resulted in over 10-fold increase in paclitaxel plasma concentrations (Fig. 3A). The elimination half-lives of paclitaxel were about 40 min in both cases, suggesting the elimination of micellar paclitaxel is rate-limited by the metabolism and excretion of free paclitaxel. The volume of distribution (V_D,SS_) of micellar paclitaxel was distinctly lower than that of free paclitaxel, indicating the distribution of the micellar drug is much restricted owing to the size of micellar nanocarriers. Accordingly, the total clearance (CL_T_) of micellar paclitaxel was about one tenth of that of the free drug, leading to the much higher circulation levels of paclitaxel over the time (area under the curve, AUC) in the body (Table 1). Similarly, the dual drug-loaded micelles also significantly elevated 17-AAG plasma concentrations (Fig. 3B) with almost 3-fold reduction in V_D,ss_ and CL_T_ (Table 1) in comparison with free 17-AAG. The pharmacokinetic parameters for free paclitaxel and 17-AAG obtained in this study were largely consistent with the literature [10], [14]. Next, we quantified the drug concentrations in tumor tissues and normal organs at 2 h. We found that the dual drug-loaded micelles yielded a 3.5-fold increase of paclitaxel and 1.7-fold increase of 17-AAG concentration in the tumor without significantly altering the drug levels in normal organs when compared with the respective free drugs (Fig. 3C and 3D). Take together, these data indicate that paclitaxel/17-AAG-loaded micelles prolong the circulation times and elevate the exposure of both drugs in the tumor.

**Table 1:** Pharmacokinetic parameters of paclitaxel/17-AAG-loaded micelles in comparison with those of the free drugs in tumor-bearing mice (mean ± SD, n = 4).

	Combined free drugs		Paclitaxel/17-AAG-loaded micelles	
	paclitaxel	17-AAG	paclitaxel	17-AAG
**t_1/2_** (min)	44.1±3.4	20.3±2.5	40.1±4.9	19.9±2.2
**AUC** (uM⋅min)	415.3±56.7	1109.8±127.3	4216.8±195.0	3316.6±174.1
**CL_T_** (ml/kg/min)	56.4±9.3	57.7±8.9	5.6±1.4	19.3±1.8
**V_D,SS_** (ml/kg)	2954.5±320.7	1445.0±211.4	311.2±36.9	468.6±75.5

Abbreviations:

t_1/2_: elimination half-life; AUC: area under the plasma drug concentration versus time curve; CL_T_: total clearance; V_D,SS_: apparent volume of distribution at the steady-state.

### NIR Imaging of ICG-labeled Micelles in the Tumor-bearing Mice

**Figure 4:**
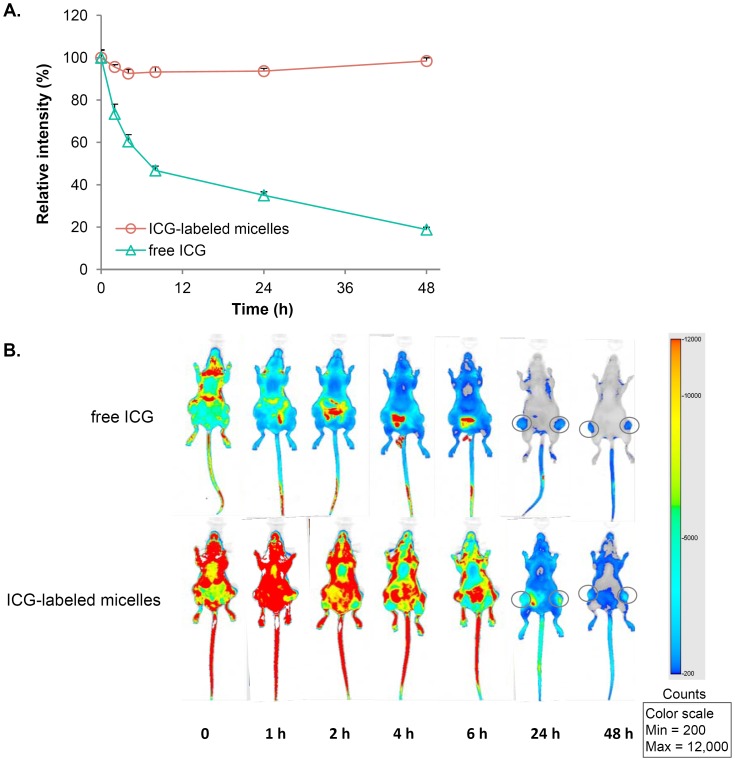
The NIR imaging of ICG-labeled micelles in nude mice bearing SKOV-3 xenografts. A, the stability of ICG at 37°C was greatly enhanced when it was loaded within PEG-DSPE/TPGS mixed micelles. In contrast, free ICG was highly unstable in aqueous solution. Results show representative data obtained from three independent experiments and are reported as the average+SD (n = 3). B, ICG-labeled micelles were i.v. injected at an ICG dose of 2 mg/kg. The control mice received an equal dose of ICG, which was freshly prepared in aqueous solution. Immediately following the injection (time 0) and at 1, 2, 4, 6, 24 and 48 h, the spectral fluorescence signals of the whole body images (800 nm channel) were obtained using the LI-COR Odyssey imaging system. Representative images are shown, n = 3 mice per group.

To further investigate the circulating and disposition characteristics of the drug-loaded micelles, we sought to incorporate an imaging dye into the micellar nanocarriers, which would stably associate with the nanocarriers and enable the real-time visualization of the formulation in the body. ICG, an FDA-approved contrast agent for vasculature imaging, has a unique NIR spectral property that is advantageous for fluorescence-based *in vivo* imaging because of its deep tissue penetration and high signal-to-noise ratio. In its free form, ICG has a poor stability in aqueous solution and is rapidly eliminated (t_1/2_<5 min) following intravenous administration [15]. We hypothesized that, by its incorporation into PEG-DSPE/TPGS mixed micelles, the aqueous stability of ICG would be greatly enhanced and its circulation time prolonged. We found that micellar ICG had less than 10% decrease in its fluorescence emission intensity when incubated in HBS at 37°C for 48 h, whereas under the identical condition that of free ICG aqueous solution decreased about 50% at 8 h and over 80% at 48 h (Fig. 4A). These stability results indicate that ICG can be stably loaded into PEG-DSPE/TPGS mixed micelles to label these nanocarriers.

Next, ICG-labeled drug-loaded PEG-DSPE/TPGS mixed micelles was evaluated in tumor-bearing mice, in comparison with free ICG at an equal dose. As shown in Fig. 4B, free ICG was rapidly cleared from the circulation, and there was only trace fluorescence signal in the body by 4−6 h except for the intestinal region, which is consistent with the literature that ICG undergoes rapid biliary secretion and consequent fecal elimination [16]. In contrast, following micellar ICG administration the fluorescence intensity throughout the body was sustained at an elevated level for at least 6 h, which was most probably emitted from ICG molecules that were associated with the micelles while circulating in the blood stream. The sustained distribution and retention of ICG in normal organs following the micelle administration was unlikely because of the following reasons: (1) the extravasation of ICG-loaded micelles across the endothelial lining of most normal organs was restricted due to the size limitation; (2) free ICG released from the micelles would be subjected to rapid elimination, as seen in free ICG-treated mice. This strongly suggests that PEG-DSPE/TPGS mixed micelles remain intact in the circulation for a prolonged period of time and provide a protective shield for the loaded molecules from elimination. Alternatively, ICG could possibly exchange between the circulating micelles and the many tissue membranes encountered in the body. ICG was most likely released from the micelles over time and was eliminated via the same pathway as free ICG. Importantly, the micellar formulation resulted in notably higher ICG accumulation in the tumor, which corroborated the tumor distribution data for paclitaxel/17-AAG-loaded micelles described above. Collectively, these results indicate that ICG can be employed as an imaging agent for monitoring PEG-DSPE/TPGS micellar delivery system *in vivo*, and confirm the enhanced accumulation of the micellar drugs in the tumor.

### Antitumor Efficacy by Paclitaxel/17-AAG-loaded Micelles in a Human Ovarian Tumor Mouse Model

**Figure 5:**
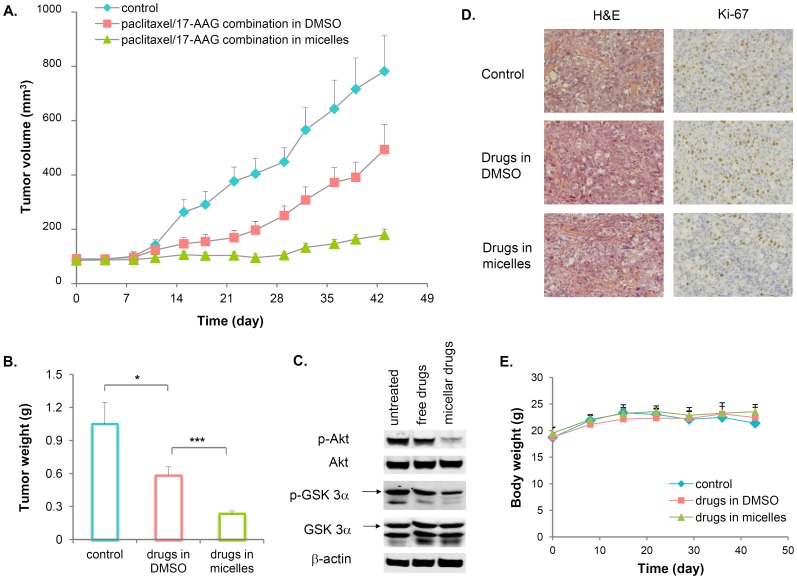
Paclitaxel/17-AAG-loaded micelles potentiate the anticancer efficacy of the drugs in nude mice bearing SKOV-3 xenografts. Mice were randomized, and treatment was initiated on day 0. The mice were i.v. dosed with the combined doses of paclitaxel (20 mg/kg) and 17-AAG (37.5 mg/kg) either as the free drugs dissolved in DMSO, or the dual drug-loaded micelles on days 0, 7 and 14. On days 3, 10 and 17, the mice in these two groups also received 17-AAG (37.5 mg/kg) as the free drug or the drug-loaded micelles. The untreated mice served as controls. A, paclitaxel and 17-AAG in their free forms significantly delayed the tumor growth but was less effective than the micellar formulation, which caused near-complete arrest of tumor growth. Starting day 22, the average tumor sizes among all three groups were significantly different (p<0.05). Each data point was the average+SE, n = 6 mice per group. B, the tumor weights on day 43 from all three groups showed drastic differences. (*, p<0.05; ***, p<0.0005) C, phosphorylation of Akt and its immediate downstream substrate GSK3α was markedly inhibited in the tumors of the micellar drug-treated mice, but remained highly activated in the tumors of the free drug-treated mice. The total levels of Akt and GSK3α were unaffected by either treatment. β-Actin was used as a loading control. The results show representative data obtained from 3 independent analyses. D, immunohistochemical staining of Ki-67 indicated a large number of proliferating tumor cells in the untreated and free drug-treated mice, but few in the micellar drug-treated mice (original magnification ×200). E, the average body weight of mice remained constant in all groups throughout the study.

In preclinical models, 17-AAG sensitizes the anticancer activity of paclitaxel by rapidly degrading key signaling proteins such as HER2, EGFR and Akt in tumor cells. It has been demonstrated that paclitaxel/17-AAG combination therapy is superior to either drug alone in retarding tumor growth [17]−[19]. Encouraged by our findings that paclitaxel/17-AAG-loaded micelles enhanced the exposure of both drugs in the tumor, we next investigated whether the micellar formulation retarded tumor growth more potently than the combined free drugs in mice bearing pre-established SKOV-3 tumor xenografts. Based on the maximum tolerated doses in cancer patients (weekly i.v. infusion of 80 mg/m^2^ for paclitaxel, twice-weekly i.v. infusion of 175 mg/m^2^ for 17-AAG) obtained from a phase I clinical trial of palcitaxel/17-AAG combination therapy [8], and because paclitaxel is known to be effective in tumor-bearing mice at 20−25 mg/kg [19], [20], a combination regimen of weekly i.v. 20 mg/kg paclitaxel and twice-weekly i.v. 37.5 mg/kg 17-AAG was chosen in our study to maintain the dose ratio between the two drugs similar to that adopted in the clinic. As shown in Fig. 5A, paclitaxel and 17-AAG in their free forms significantly delayed the tumor growth, but the mice experienced rapid tumor growth shortly after a 3-week treatment period ended. In contrast, the micellar formulation of paclitaxel and 17-AAG caused near-complete arrest of tumor growth. The drastic differences in the tumor weight among the treatment groups on day 43 was shown in Fig. 5B. The sustained tumor growth inhibition by micellar paclitaxel and 17-AAG was accompanied by marked downregulation of Akt signaling in the tumor tissues, reflected by the reduced levels of p-Akt and p-GSK3α, an immediate downstream substrate of Akt (Fig. 5C). On the other hand, Akt signaling remained highly activated in the tumors of the free drug-treated mice (Fig. 5C). Immunohistochemistry staining of the tumor tissues with Ki-67, a cell proliferation marker, revealed that the tumor cells in the micellar drug-treated mice underwent much less proliferation, whereas there was little difference between the tumors of the untreated and free drug-treated groups in term of their proliferation status (Fig. 5D). The Ki-67 staining index was about 30**–**35% in both the untreated and free drug-treated tumors, and below 10% in the micellar drug-treated tumors. These results clearly indicate that the micellar formulation of paclitaxel and 17-AAG blocks the tumor growth for a prolonged period of time, and potentiates the anticancer efficacy of paclitaxel/17-AAG combination therapy. There was little overall toxicity with either the free or micellar drugs since the average body weight of mice remained constant throughout the study (Fig. 5E). To examine the potential cellular toxicities associated with the drug-loaded PEG-DSPE/TPGS mixed micelles against the major organs, the hepatic and renal functional tests upon completion of the efficacy study are warranted in future studies.

### Effect of Paclitaxel/17-AAG Combination Therapy on the Tumor Metabolome

**Figure 6:**
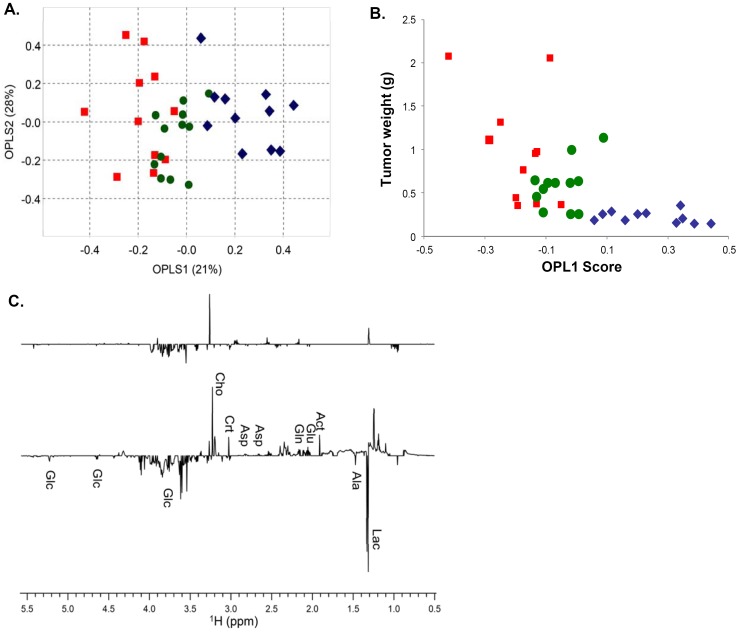
Metabolomic analysis of the mouse tumor extracts. A, scores plot of the OPLS-DA model for the untreated and treated tumors, which shows the group separation among the untreated (square), free drug-treated (circle) and micellar drug-treated (diamond) tumors. The percentages of explained variation modeled for the first two components (OPLS1 and OPLS2) are displayed on the axes. B, there was no clear correlation between the OPLS1 scores and their corresponding tumor weights. C, Student’s t-test filtered difference NMR spectra (p<0.05) of tumors treated with paclitaxel/17-AAG combination in the free forms (top) and in the micellar formulation (bottom). The positive peaks correspond to the metabolites increased in concentration relative to the untreated tumors, whereas the negative peaks are from the metabolites decreased. The identified metabolites are: Glc, glucose; Cho, choline; Crt, creatine; Asp, aspartate; Gln, glutamine; Glu, glutamate; Ala, alanine; Lac, lactate.

To further understand the mechanism that account for the improved anticancer efficacy of paclitaxel/17-AAG-loaded micelles, we carried out ^1^H NMR spectroscopy study to assess the metabolome of the tumor tissues harvested on day 43. As shown in Fig. S1 and Fig. 6A, the PCA and OPLS-DA analyses allowed for clear group separation among the untreated, free drug-treated and micellar drug-treated tumors. The OPLS-DA model was validated using a training set (80% of the data) and a prediction set (the remaining 20% of the data), which showed 100% accuracy in the group prediction (Fig. S2). These results indicate that, the tumor cells in the different treatment groups undergo significantly altered metabolic activities. To investigate whether the metabolomic alterations were dependent on the tumor size, the OPLS1 score of each tumor was plotted against the tumor weight (Fig. 6B). We found that there was no clear correlation between the OPLS1 scores and the corresponding tumor weights, and a narrow range of tumor weights (e.g. 0.2–0.5 mg) possessed a wide range of the OPLS1 scores (-0.2–0.4). These results indicate that even for the tumors with similar sizes, their metabolomic profiles vary significantly owing to the different treatment the mice received.

In the next step, we generated two t-test filtered difference NMR spectra in search for the metabolites responsible for the group separation (Fig. 6C). It is important to note that only the metabolites with ^1^H NMR peaks that passed a univariant t-test with a p-value <0.05 were shown in the difference spectra. The resulting t-test filtered difference spectrum displayed peaks above the baseline corresponding to the metabolites that were increased upon the drug treatment, and the peaks below the baseline corresponding to the decreased metabolites. Since both difference spectra were displayed using the same Y-axis scale, the overall magnitude of change in the metabolite levels was proportional to the peak intensity in each spectrum. A total of 9 metabolites were the major contributors to the group separation and were related to glucose, glutamine and phospholipid metabolism. Compared with the untreated tumors, we found significant decrease in glucose, lactate and alanine with simultaneous increase in glutamine, glutamate, aspartate, choline, creatine and acetate levels in the micellar drug-treated tumors (Fig. 6C bottom spectrum). This is consistent with the changes indicated by the OPLS-DA loading plot (Fig. S3). In the meantime, treatment with free paclitaxel and 17-AAG only caused to a lesser extent an increase in choline and a decrease in glucose (Fig. 6C top spectrum). Together, these results reveal distinct metabolomic alterations that correlated with the enhanced anticancer efficacy of paclitaxel/17-AAG-loaded micelles.

## Discussion

Among the numerous intravascular nanvectors that are currently being pursued in the preclinical and clinical studies, block copolymer micelles are recognized as promising drug delivery systems for poorly water-soluble drugs [21]. Self-assembled from the amphiphilic copolymers, polymeric micelles are characterized by a hydrophobic core that can efficiently solubilize hydrophobic drug molecules, and a hydrophilic corona that sterically stabilizes the micellar structure in the aqueous milieu. Genexol-PM, a Cremophor EL-free formulation for paclitaxel that is undergoing phase II and III clinical trials, employs polyethylene glycol-*block*-poly(D,L-lactic acid) (PEG-PLA) micelles to solubilize and deliver paclitaxel. Obviation of Cremophor EL-related toxicity allows Genexol-PM to be administered to cancer patients at higher doses than the conventional Cremophor EL-based paclitaxel formulation without a significant increase in toxicities [22]. PEG-PLA micelles have recently been explored for the combined delivery of multiple drugs including paclitaxel and 17-AAG [6]. It is nevertheless questionable whether the drug-loaded PEG-PLA micelles could accumulate in the tumor via the EPR effect, because of the very rapid drug release from PEG-PLA micelles in the circulation [23].

In search for an alternative formulation for the conbined delivery of paclitaxel and 17-AAG, we aimed to identify a drug carrier system that is not only capable of solubilizing the drugs, but also enhancing drug delivery to the tumor. We have previously demonstrated that PEG-DSPE/TPGS mixed micelles can greatly increase the aqueous solubility of paclitaxel and 17-AAG, and release the loaded drugs in serum at much slower rates in comparison with PEG-PLA micelles [9]. In the current study, the disposition and antitumor efficacy of paclitaxel/17-AAG-loaded PEG-DSPE/TPGS mixed micelles were evaluated in tumor-bearing mice. As shown in our pharmacokinetic study, the dual drug-loaded micelles markedly extended the circulation times of both drugs and elevated drug concentrations in the tumor compared with the equivalent doses of the free drugs, which was further confirmed by the NIR imaging of ICG-labeled micelles in the tumor-bearing mice over a 48-h period. These results strongly suggest that in the circulation a significant proportion of PEG-DSPE/TPGS micelles retain the loaded paclitaxel and 17-AAG and enhance drug delivery to the tumor tissue via the EPR effect. Following the extravasation into the tumor, the drug-loaded PEG-DSPE/TPGS mixed micelles may undergo two possible processes: (1) the micelles could be taken up by the tumor cells via endocytosis, which subsequently release the drugs inside the cytoplasm; (2) the drugs could be first released from the micelles, and then penetrate into the tumor cells in the free forms. Owing to the inefficient nature of the first process without a targeting ligand on the micellar surface, the second process is likely to be the dominant route for these micellar drugs to gain intracellular access and exert antitumor activities. In line with the improved pharmacokinetic characteristics associated with the micellar formulation, our efficacy study clearly demonstrated that paclitaxel/17-AAG-loaded micelles elicited more potent and persistent tumor growth arrest than the combined free drugs at the equivalent doses. With paclitaxel and 17-AAG as model hydrophobic anticancer drugs, these findings demonstrate the potential of PEG-DSPE/TPGS mixed micelles as a drug carrier system to eliminate the use of toxic organic solubilizers, deliver multiple drugs concurrently and achieve enhanced anticancer efficacy.

Our metabolomic analysis of the tumor samples provides *in vivo* mechanistic insight into the antitumor efficacy of paclitaxel/17-AAG combination therapy. Compared with the metabolomic profiles of the untreated tumors, the micellar formulation resulted in distinct and significant metabolomic alterations in the tumor tissues, which were associated with the attenuated Akt signaling and the low Ki-67 proliferation index in the tumors. In contrast, the metabolomic differences between the free drug-treated and untreated tumors were much less prominent. Even though the tumor sizes in the free drug-treated mice were significantly smaller than those of the untreated mice, the inhibition on tumor growth caused by free paclitaxel and 17-AAG was transient, and the tumor cells underwent rapid proliferation similar to those in the untreated mice on day 43, as indicated by their comparable Ki-67 index. These results reveal an intriguing linkage between the metabolic activities of the tumor cells and their proliferation status.

Tumor cells display a robust and reprogrammed metabolome that supports rapid synthesis of macromolecules including lipids, proteins, and nucleosides, which are all required for the proliferation of the tumor cells [24], [25]. A major hallmark of tumor cell metabolism is the rapid aerobic glycolysis and the dependency on the glycolytic pathway for ATP generation, the so-called Warburg effect, characterized by the increased glucose uptake and lactate/alanine production [26]. The results from our comparative metabolomic profiling strongly suggest that paclitaxel/17-AAG-loaded micelles normalize glucose consumption in the tumor by reducing glucose uptake and blocking aerobic glycolysis, leading to significantly decreased levels of glucose in the tumor, as well as lactate and alanine which are the end products of glycolysis. The molecular mechanism accountable for this observation is likely in part due to the blockage of Akt signaling by 17-AAG. As a master regulator of tumor metabolism, activation of Akt signaling pathways promotes glucose uptake by upregulating glucose transporters, and enhances glycolysis by modulating key enzymes [27]. 17-AAG has been shown to abrogate Akt activation via Hsp90 inhibition in a dose- and time-dependent manner, which sensitizes the tumor cells to the anticancer activity of paclitaxel [18]. The results from our Western blot analysis on the tumor tissue samples clearly showed that Akt signaling was pronouncedly suppressed in the tumors treated with paclitaxel/17-AAG-loaded micelles, even though the treatment was ended three weeks earlier.

In addition, paclitaxel/17-AAG-loaded micelles also altered glutamine metabolism in the tumor. Glutamine is another major cancer cell energy and anabolic substrate that, after being converted into glutamate, it enters the tricarboxylic cycle and is metabolized to aspartate, which contributes to the nucleoside synthesis [28]. The increased tumor levels of glutamine, glutamate and aspartate resulting from the micelle treatment strongly imply that *de novo* synthesis of nucleic acids is attenuated in the tumor cells. Furthermore, the accumulation of choline in the tumor upon the micelle treatment strongly suggests a slowed-down production of cell membranes, where choline functions as a key precursor for phospholipid synthesis [29]. Together, the metabolomic alterations observed in the micellar drug-treated tumors are well correlated with the potent antiproliferative activity of the treatment. Because PEG-DSPE and TPGS do not possess anticancer activity per se [9], [30], it is unlikely the micelle components alone could be responsible for the pronounced metabolomic differences between the free and micellar drug-treated tumors. Rather, paclitaxel/17-AAG-loaded micelles caused a more profound and lasting impact on the tumor metabolome by significantly increasing the drug exposure in the tumor, leading to superior anticancer efficacy. These metabolomic alterations provide mechanistic explanations for tumor growth retardation observed in the mice treated with paclitaxel/17-AAG-loaded micelles, which may be used as pharmacodynamic markers to monitor and predict the effectiveness of paclitaxel/17-AAG combination therapy in the clinical setting. Molecular imaging technologies such as positron emission topography and magnetic resonance spectroscopic imaging can potentially be utilized to discriminate some of these metabolomic alterations noninvasively *in vivo*.

In summary, we have demonstrated in the current wok a safe and efficacious nano-sized formulation for combined paclitaxel/17-AAG delivery. With a broad applicability to deliver hydrophobic anticancer drugs *in vivo,* this drug delivery system is highly translatable to the clinic because of the ease of the formulation preparation without any chemical conjugation, and the established safety profiles of PEG-DSPE and TPGS copolymers, both of which are FDA-approved pharmaceutical excipients. The metabolomic signatures uncovered in our ^1^H NMR study could be used to predict favorable drug response to paclitaxel/17-AAG combination therapy.

## Supporting Information

Figure S1 **Two-component PCA scores plot of the tumor tissue extracts, which was used to examine the data set for outliers.** All samples were within the Hotelling’s T^2^ test at the 95% confidence, indicating that there was no outlier. Red squares, green circles and blue diamonds represent the untreated, free drug-treated, and micellar drug-treated tumor samples, respectively. The percentages of explained variation for the first two components (PC1 and PC2) are displayed on the axes.
(DOC)

Figure S2 **Validation of the OPLS-DA model.** The OPLS-DA model was first generated with a training set (∼80% of the data) and the model was then used to predict OPLS1 scores of the prediction set (the remaining ∼20% of the data). All predicted scores (grey triangles) were located within their own groups, indicating that the OPLS-DA model has a good predictability. Red squares, green circles and blue diamonds represent the untreated, free drug-treated, and micellar drug-treated tumor samples, respectively.
(DOC)

Figure S3 **Loadings plot of the OPLS-DA model for the untreated and treated tumor samples, which was used to identify the metabolites responsible to the group separation in the scores plot.** The metabolic profiles of the treated tumors were characterized with decreased levels of glucose (Glc), alanine (Ala), and lactate (Lac) and elevated levels of acetate (Act) and choline (Cho).
(DOC)
