# Supplementary figures and images for: Combined Delivery of Paclitaxel and Tanespimycin via Micellar Nanocarriers: Pharmacokinetics, Efficacy and Metabolomic Analysis
Source: PLoS One. 2013 Mar 7;8(3):e58619. doi: 10.1371/journal.pone.0058619 (PMC3591361; doi:10.1371/journal.pone.0058619)

**Supplementary Figure S1.**

**
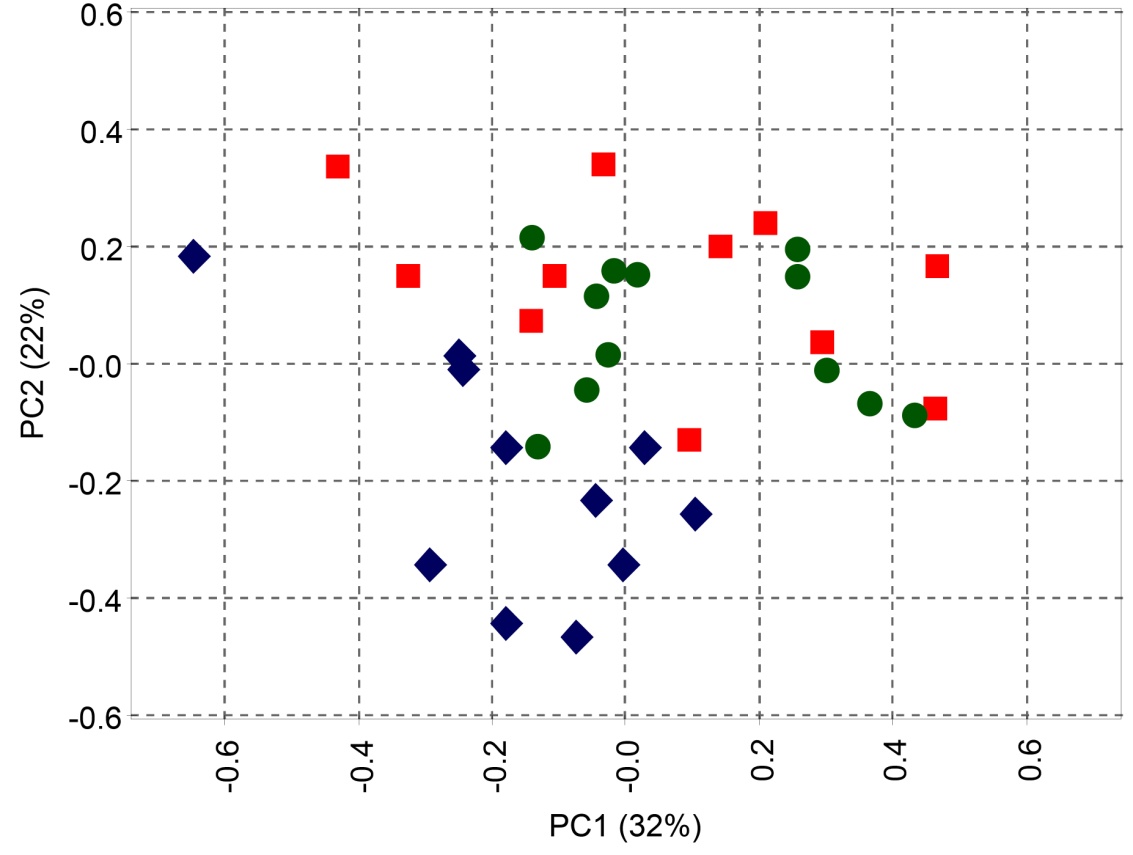
**

Supplement: Figure S1 — Two-component PCA scores plot of the tumor tissue extracts, which was used to examine the data set for outliers. All samples were within the Hotelling’s T2 test at the 95% confidence, indicating that there was no outlier. Red squares, green circles and blue diamonds represent the untreated, free drug-treated, and micellar drug-treated tumor samples, respectively. The percentages of explained variation for the first two components (PC1 and PC2) are displayed on the axes. (DOC) [file pone.0058619.s001.doc]

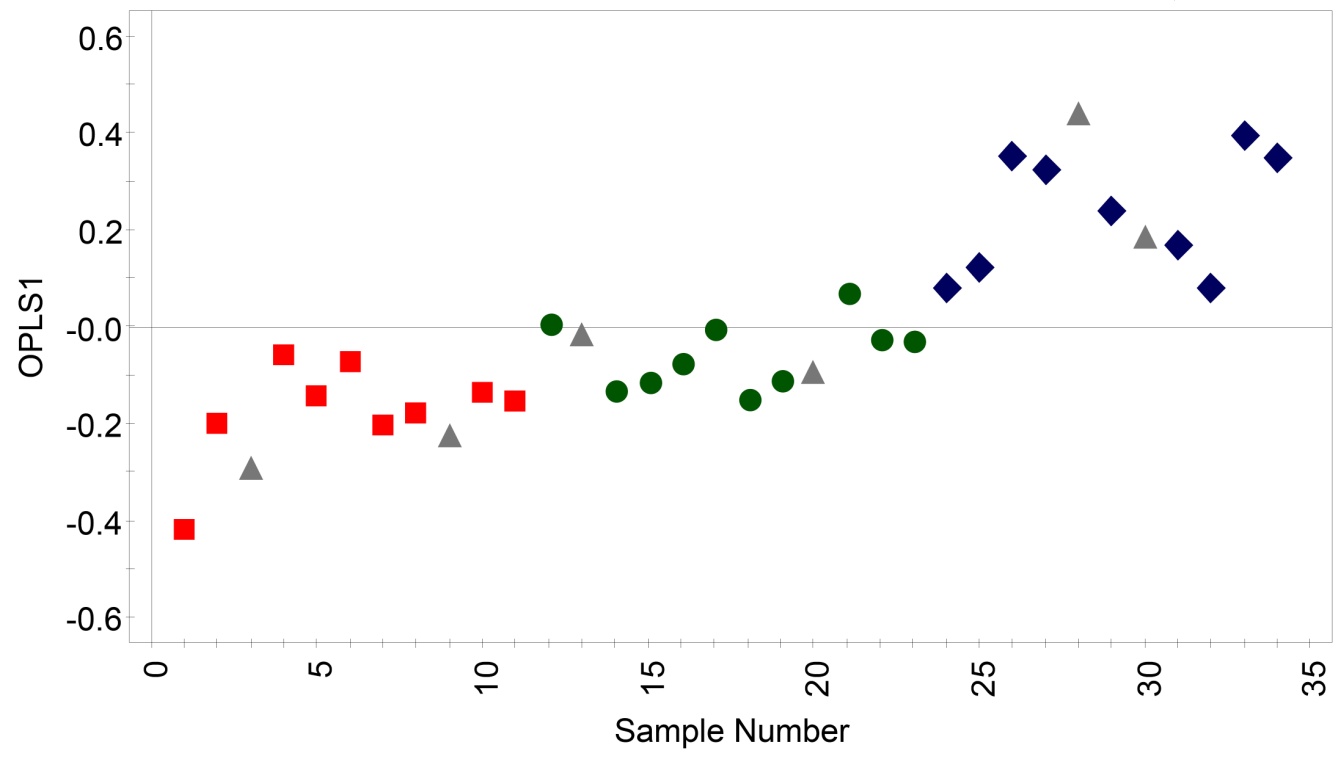
**Supplementary Figure S2.**

Supplement: Figure S2 — Validation of the OPLS-DA model. The OPLS-DA model was first generated with a training set (∼80% of the data) and the model was then used to predict OPLS1 scores of the prediction set (the remaining ∼20% of the data). All predicted scores (grey triangles) were located within their own groups, indicating that the OPLS-DA model has a good predictability. Red squares, green circles and blue diamonds represent the untreated, free drug-treated, and micellar drug-treated tumor samples, respectively. (DOC) [file pone.0058619.s002.doc]

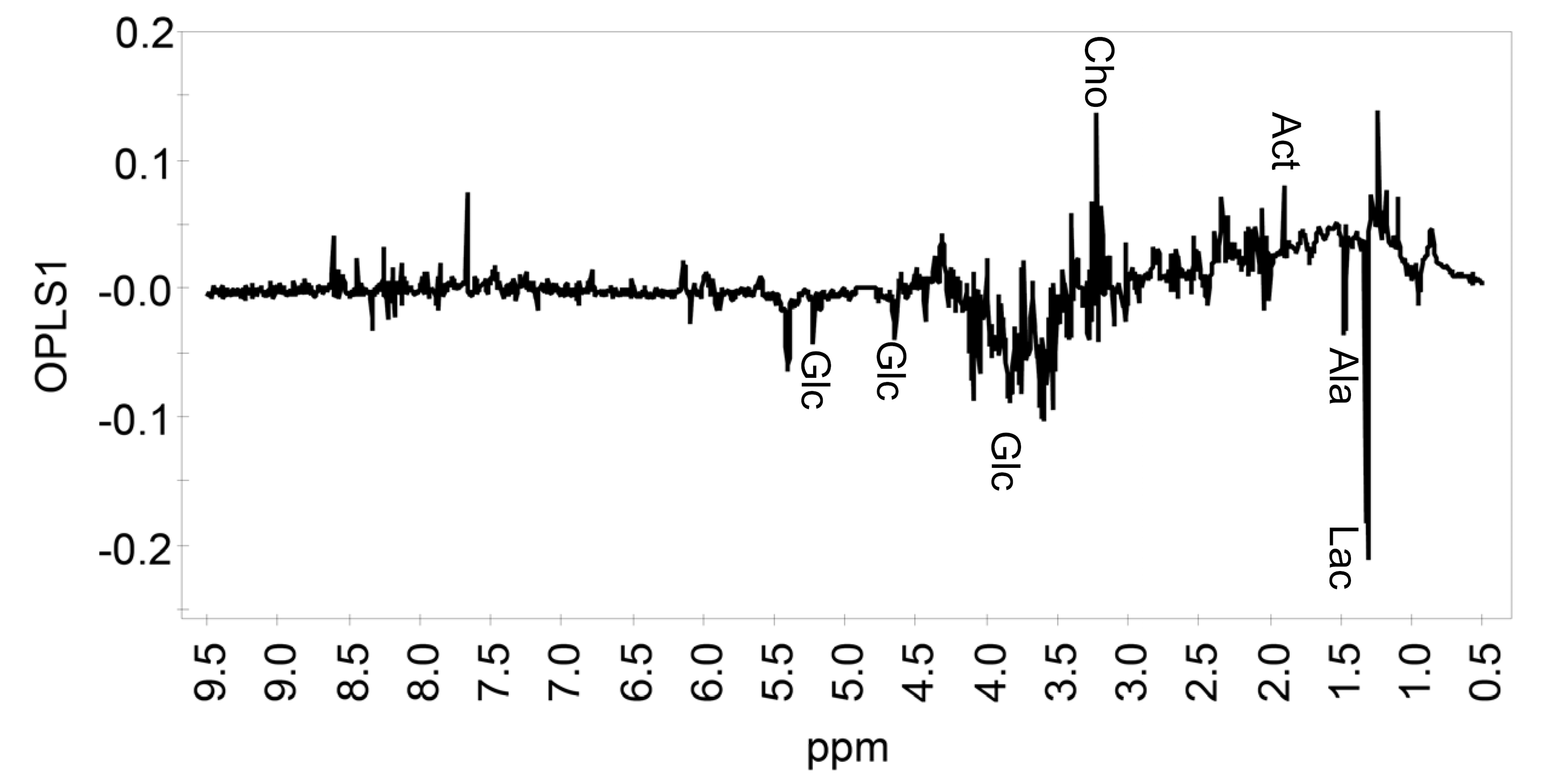
**Supplementary Figure S3.**

Supplement: Figure S3 — Loadings plot of the OPLS-DA model for the untreated and treated tumor samples, which was used to identify the metabolites responsible to the group separation in the scores plot. The metabolic profiles of the treated tumors were characterized with decreased levels of glucose (Glc), alanine (Ala), and lactate (Lac) and elevated levels of acetate (Act) and choline (Cho). (DOC) [file pone.0058619.s003.doc]
